# Mapping B-Cell Epitopes for the Peroxidoxin of *Leishmania (Viannia) braziliensis* and Its Potential for the Clinical Diagnosis of Tegumentary and Visceral Leishmaniasis

**DOI:** 10.1371/journal.pone.0099216

**Published:** 2014-06-12

**Authors:** Daniel Menezes-Souza, Tiago Antônio de Oliveira Mendes, Ronaldo Alves Pinto Nagem, Thaís Teodoro de Oliveira Santos, Ana Luíza Teixeira Silva, Marcelo Matos Santoro, Silvio Fernando Guimarães de Carvalho, Eduardo Antônio Ferraz Coelho, Daniella Castanheira Bartholomeu, Ricardo Toshio Fujiwara

**Affiliations:** 1 Departamento de Parasitologia, Instituto de Ciências Biológicas, Universidade Federal de Minas Gerais, Belo Horizonte, Brazil; 2 Departamento de Bioquímica e Imunologia, Universidade Federal de Minas Gerais, Belo Horizonte, Brazil; 3 Centro de Ciências Biológicas e da Saúde, Universidade Estadual de Montes Claros, Montes Claros, Brazil; 4 Programa de Pós-Graduação em Ciências da Saúde. Infectologia e Medicina Tropical, Faculdade de Medicina and Departamento de Patologia Clínica, COLTEC, Universidade Federal de Minas Gerais, Belo Horizonte, Minas Gerais, Brazil; Technion-Israel Institute of Technology Haifa 32000 Israel, Israel

## Abstract

The search toward the establishment of novel serological tests for the diagnosis of leishmaniasis and proper differential diagnosis may represent one alternative to the invasive parasitological methods currently used to identify infected individuals. In the present work, we investigated the potential use of recombinant peroxidoxin (*r*Peroxidoxin) of *Leishmania (Viannia) braziliensis* as a potential antigen for the immunodiagnosis of human tegumentary (TL) and visceral leishmaniasis (VL) and canine visceral leishmaniasis (CVL). Linear B-cell epitope mapping was performed to identify polymorphic epitopes when comparing orthologous sequences present in *Trypanosoma cruzi*, the agent for Chagas disease (CD), and the *Homo sapiens* and *Canis familiaris* hosts. The serological assay (ELISA) demonstrated that TL, VL and CVL individuals showed high levels of antibodies against *r*Peroxidoxin, allowing identification of infected ones with considerable sensitivity and great ability to discriminate (specificity) between non-infected and CD individuals (98.46% and 100%; 98.18% and 95.71%; 95.79% and 100%, respectively). An *r*Peroxidoxin ELISA also showed a greater ability to discriminate between vaccinated and infected animals, which is an important requirement for the public campaign control of CVL. A depletion ELISA assay using soluble peptides of this B-cell epitope confirmed the recognition of these sites only by *Leishmania*-infected individuals. Moreover, this work identifies two antigenic polymorphic linear B-cell epitopes of *L. braziliensis*. Specific recognition of TL and VL patients was confirmed by significantly decreased IgG reactivity against *r*Peroxidoxin after depletion of peptide-1- and peptide-2-specific antibodies (peptide 1: reduced by 32%, 42% and 5% for CL, ML and VL, respectively; peptide-2: reduced by 24%, 22% and 13% for CL, ML and VL, respectively) and only peptide-2 for CVL (reduced 9%). Overall, *r*Peroxidoxin may be a potential antigen for the immunodiagnosis of TL, VL or CVL, as it has a higher agreement with parasitological assays and is better than other reference tests that use soluble *Leishmania* antigens for diagnosing CVL in Brazil (EIE-LVC, Bio-manguinhos, FIOCRUZ).

## Introduction

Leishmaniasis is prevalent in 98 countries, with an incidence estimated at 1.5 to 2 million cases per year [1]. Diagnostic investigations for individual cases include the search of the suggestive history and clinical features associated with a positive Montenegro skin test (MST), identification of amastigotes by histology or direct microscopy, the growth of promastigotes in culture or PCR amplification of the parasite DNA [2]. Despite the high specificity, these methods have several limitations, such as variation in sensitivity because the parasite distribution in the tissue is not homogeneous and the reliance on invasive procedures and strict conditions for specimen collection that depends on complex structures and laboratory procedures—facts that hinders the employment of these methods in large-scale epidemiological studies [3].

In this context, antigen- or antibody-based detection tests, such as enzyme-linked immunoassays (ELISA) have advantages, as they do not require special specimen-transport conditions and can be performed in local laboratories within 3–4 hours and can be used as important tools for the diagnosis and epidemiological study of leishmaniasis [4]. Currently, the search toward the establishment of novel serological tests for an accurate differential and the precise diagnosis may represent one of the most relevant challenges for the control and possible eradication of tegumentary (TL) and visceral (VL) leishmaniasis. The parasitological techniques commonly employed are invasive, time-consuming, and inappropriate for epidemiological surveillance [5]. On the other hand, the ELISA has proved to be a sensitive method and suitable for epidemiological surveys; however, cross-reactivity with other infections such as American trypanosomiasis, as well as *Leishmania* vaccines, is often reported [6]–[8].

Several studies have also employed antigens of dermotropic *Leishmania* species to immunodiagnostics and vaccines with greater antigenicity and immunogenicity against viscerotropic species, such as *L. infantum* [9]–[11]. In the present work, we evaluated the potential of the predicted protein peroxidoxin of *L. braziliensis*, which is a highly conserved protein in the *Leishmania* genus, as new targets for the serological diagnosis of TL, VL and canine visceral leishmaniasis (CVL). The strategy used to identify specific targets for the ELISA was to map polymorphic linear B-cell epitopes present in proteins present in the predicted proteome of *L. braziliensis* and to assess the cross-reactivity with other infections combining the proteome data from these parasites [12]. Through bioinformatic analysis, we selected peroxidoxin by presenting two highly antigenic and polymorphic linear B-cell epitopes when compared to orthologs present in *Homo sapiens*, *Canis familiaris* and *Trypanosoma cruzi* proteomes. TL and VL patients showed high levels of antibodies against *r*Peroxidoxin, allowing identification of the cutaneous, mucosal and visceral clinical forms with considerable sensitivity and specificity. The specific recognition was confirmed by a significantly decreased IgG reactivity against *r*Peroxidoxin after the depletion of peptide-specific antibodies by synthetic peptides of predicted epitopes using sera from TL and VL patients compared with chagasic patients and non-infected individuals. Our results also demonstrated that dogs infected by *L. infantum* showed high levels of antibodies against *r*Peroxidoxin, allowing identification with high sensitivity CVL. These results confirmed the high antigenicity and specificity of peroxidoxin as a potential antigen for the immunodiagnosis of TL, VL and CVL. Additionally, when evaluating animals infected with other relevant canine pathogens, such as *Trypanosoma cruzi*, an *r*Peroxidoxin ELISA demonstrated a greater ability to discriminate dogs suffering from VL among these infections, compared with a reference test for CVL diagnosis in Brazil, EIE-LVC (Bio-manguinhos, FIOCRUZ). The *r*Peroxidoxin ELISA also exhibited a greater ability to discriminate between vaccinated and infected animals, which is an important requirement for the public campaign control of CVL. Finally, the present study demonstrated that *r*Peroxidoxin is one of the target molecules that might be used as a potential antigen for immunodiagnosing different *Leishmania* species.

## Materials and Methods

### Ethics statement and human and dog sera samples

All samples used were anonymized and obtained from the sera bank of the Laboratory of Immunology and Genomic of Parasites, Federal University of Minas Gerais. Approval to use the samples was obtained from the Human Research Ethics Committee (Protocol CAAE – 00842112.2.0000.5149) and the Committee on Ethics of Animal Experimentation from the Federal University of Minas Gerais (protocol #44/2012).

The human sera panel consisted of 65 samples from TL patients infected with *L. braziliensis* and presenting cutaneous (CL, n = 45) or mucosal (ML, n = 20) clinical manifestations, from the Centro de Referência em Leishmaniose (Januária, Minas Gerais State, Brazil), and 55 samples from visceral leishmaniasis patients infected with *L. infantum*, from the University Hospital (Montes Claros, Minas Gerais State, Brazil). The infection was confirmed by the microscopic analysis of biopsies from the cutaneous lesion (TL) or the bone marrow aspirate (VL), followed by specific PCR assays for kDNA from the *Leishmania* parasite [13]. These individuals were known to be un-infected with *T. cruzi*. Information on the clinical evaluations and PCR results were obtained from the patients' medical records. To evaluate the cross-reactivity with Chagas disease, sera from chronic chagasic patients (CD, n = 20) with infection confirmed by hemoculture or by both the Chagatest recombinant ELISA v.3.0 kit (Wiener Lab, Argentina) and the Chagatest hemagglutination inhibition (HAI) assay (Wiener Lab), were used in this study. Sera from healthy Brazilian individuals (CT, n = 50) residing in an area non-endemic for TL and CD (Belo Horizonte, Minas Gerais State, Brazil) were used as a negative control in these assays. The resulting OD values were compared with those obtained with the panel of TL and VL samples.

Male and female beagle dogs from a non-endemic area for VL and with negative results for *Leishmania* in tissue smears (bone marrow) were considered to be non-infected and were used as the control group (CD, n = 51). *Leishmania*-infected dog sera (CVL, n = 95) were obtained from the area endemic for CVL in Minas Gerais State of Southeast Brazil. The main inclusion criterion for CVL sera samples used in this study was parasitological positivity for *L. infantum*, confirmed by the microscopic analysis of bone marrow aspirates. Samples from dogs experimentally infected with *T. cruzi* (TC, n = 16) or immunized with commercial vaccines Leishmune (Fort Dodge) (LM, n = 6) or Leish-tec (Hertape Calier) (LT, n = 16), but parasitologically negative for *Leishmania*, were included in this study to evaluate possible cross-reactivity.

### In silico prediction of linear B-cell epitopes

Linear B-cell epitopes were predicted for the peroxidoxin protein of *L. braziliensis* (TritripDB ID [14]: LbrM.23.0050) using the Bepipred 1.0 program with a cutoff of 1.3 [15]. A Pfam (Version 26.0) search [16] was performed using HMMer via the Pfam server at http://pfam.sanger.ac.uk/. A BLASTp [17] search was performed against GeneDB (http://www.genedb.org/) to retrieve peroxidoxin from *T. cruzi*, *Homo sapiens* and *Canis familiaris* using *L. braziliensis* peroxidoxin as a query. Multiple alignment of peroxidoxin was performed by the ClustalX 2.0. program [18], using default parameters [12].

### Soluble *Leishmania braziliensis* Antigen (SLbA)

Soluble *Leishmania* antigen was prepared from *L. (Viannia) braziliensis* (SLbA) (MHOM/BR/75/M2904) and *L. (Leishmania) infantum* (SLiA) (MHOM/BR/1972/BH46) stationary phase promastigotes maintained in Schneider's Insect medium (Sigma-Aldrich) supplemented with 10% inactivated fetal bovine serum, 100 U/ml penicillin and 100 µg/ml streptomycin (Gibco). The parasites were initially submitted to 3 cycles of freezing (liquid nitrogen) and thawing (42°C), followed by ultrasonication (Ultrasonic processor, GEX600), alternating 10 cycles of 30 sec on/off sonication in an ice–water bath at 35 MHz and then centrifuged at 6,000×*g*, 4°C for 15 min. The supernatant containing SLbA was collected and the protein content quantified using the Pierce BCA Protein Assay (Thermo Scientific). We decided to use the soluble antigen of *L. braziliensis* for immunodiagnosing VL because the recombinant protein was derived from this species, and it would not be appropriate for comparison with the soluble antigen of another species, as described previously [19]. However, data from ELISA using soluble *L. infantum* antigen for diagnosis of VL is shown in Figure S1.

### Cloning, protein expression and purification

The primers used to amplify the peroxidoxin gene from the *Leishmania* genomic DNA were Peroxidoxin-Forward, 5′ **GCTAGC** ATGCTCCGTCGTCTTGCT, and Peroxidoxin-Reverse, 5′ **AAGCTT** TCACATATTCTTCTCAAAAAATTCGC. The sites for restriction enzymes (*Nhe*I and *Hind*III, respectively), added to facilitate cloning, are shown in bold. The amplified DNA fragments were excised from the gel, purified and linked into *p*GEM-T Vector Systems (Promega, USA). Recombinant plasmid *p*GEM-peroxidoxin was used to transform *Escherichia coli* XL1-Blue (Phoneutria, Brazil) competent cells. Positive transformants were tested by restriction analysis with *Nhe*I and *Hind*III, and those presenting the peroxidoxin gene were propagated and used for constructing the expression vector. DNA fragments obtained from digestion of *p*GEM-peroxidoxin with *Nhe*I and *Hind*III were ligated into *p*ET28a-TEV [20]. Electrocompetent *E. coli* BL21 Arctic Express (DE3) (Agilent Technologies, USA) cells were transformed by electroporation using a MicroPulser Electroporation Apparatus (Bio-Rad Laboratories, USA) with the recombinant plasmid *p*ET28a-TEV-peroxidoxin. Gene insertion was confirmed by colony PCR and sequencing using T7 primers (Macrogen, South Korea).

Protein expression and purification were performed according to previous studies [21]. Briefly, the expression was induced in transformed *E. coli* by the addition of IPTG to a final concentration of 1.0 mM, and the culture was incubated for 24 h, at 12°C and 200 rpm.min^−1^. The cells were ruptured by sonication, the debris was removed by centrifugation, and the recombinant protein was purified onto a HisTrap HP affinity column connected to an ÄKTAprime chromatography system (GE Healthcare, USA). The eluted fractions containing the *r*Peroxidoxin (227 amino acids, 25.3 kDa) were concentrated in Amicon ultra 15 Centrifugal Filters 10,000 NMWL (Millipore, Germany) and further purified on a Superdex 200 gel-filtration column (GE Healthcare Life Sciences, USA).

### Soluble peptide synthesis

The soluble peptides were manually synthesized by Fmoc chemistry in the solid phase on a 30-µmol scale [22]. Briefly, the Fmoc-amino acids were activated with a 1∶2 solution of Oxyme and DIC. The active amino acids were incorporated into Rink amide resin with a substitution degree of 0.61. Fmoc deprotection was then performed using 25% 4-methylpiperidine. These steps were repeated until the synthesis of each peptide was complete. The peptides were deprotected and released from the resin by treatment with a solution of 9.4% trifluoroacetic acid, 2.4% water, and 0.1% triisopropylsilane. The peptides were precipitated with cold diisopropyl ether and purified by Superdex Peptide (30 pg) (GE Healthcare, USA). The peptides were obtained with at least 90% purity, as confirmed by mass spectrometry using MALDI-TOF-TOF Autoflex III equipment (Bruker Daltonics, USA). Instrument calibration was achieved using Peptide Calibration Standard II (Bruker Daltonics, USA) as a reference, and a-cyano-4-hydroxycinnamic acid was used as the matrix.

### Serological assay and depletion ELISA

Antigenicity was evaluated by the determination of antibodies against *r*Peroxidoxin according to the conventional ELISA. The antibody ELISA and protein quantity were optimized to obtain the best signal-to-noise ratio and to develop a reproducible and robust assay that was capable of capturing antibodies over a biologically relevant assay range (data not showed). *r*Peroxidoxin was coated onto 96-well microplates (Nalge Nunc Intl., USA) overnight at 2–8°C at a concentration of 500 ng/well for TL and VL, 50 ng/well for CVL and *r*Peroxidoxin and 100 ng/well for SLbA. The plates were blocked with 100 µL of 5% PBS-BSA for 1 h at 37°C and treated successively with 1∶100 dilutions of the human serum samples for 1 h at 37°C. Peroxidase-labeled antibodies specific to human or dog IgG (Sigma-Aldrich, USA) were diluted at 1∶5,000 and added for 1 h at 37°C. The wells were washed, and a TMB substrate (Sigma-Aldrich, USA) in citrate buffer containing hydrogen peroxide was added. The plates were incubated for 30 min in the dark. The reactions were stopped by the addition of 4 N H_2_SO_4_, and the absorbance read at 450 nm on automatic microplate reader (Versamax, Molecular Devices, USA). Each sera sample was assayed in duplicate. The results of the ELISA using *r*Peroxidoxin as antigens were compared with SLbA. The results of the ELISA using *r*Peroxidoxin as antigens in dogs were compared with the immunoassay EIE-LVC kit (FIOCRUZ-Bio-Manguinhos, Brazil), which is the test currently recommended by the Brazilian Ministry of Health for screening seroreactive animals [23]. The assays were conducted according to the manufacturer's instructions.

The depletion ELISAs were performed as previously described [24]. First, the peptides were tested for the ability to detect TL, VL and CVL (data not shown). Briefly, flat-bottom plates (Costar, USA) were coated overnight with 2 µg/well of peptide-1 (VRDPAPQFS) and peptide-2 (TPGKPTLDT), then washed and blocked as described. A pool of sera from ten individuals per group (randomly selected) were added to the plates at a 1∶100 dilution and incubated for 1 h at 37°C. In the following step, the sera were transferred to plates coated overnight with *r*Peroxidoxin (500 ng/well for TL and VL; 50 ng/well for CVL) after appropriate washing and blocking, and the ELISAs were performed as described previously. We also performed the depletion assay using peptide unrelated (TYHGIPCDSGRNCKKYENF) as reaction control. The peptides were first tested for the ability to detect TL, VL and CVL.

### Statistical analysis

The lower limit of positivity (cut-off) for *r*Peroxidoxin and SLbA was established for optimal sensitivity and specificity using the Receiver Operator Curve (ROC curve). The cut-off was chosen based on the point that provides the maximum of the sum of the sensitivity and specificity [25]. The performance of each test was evaluated according to the sensitivity (Se), specificity (Sp), positive predictive value (PPV), negative predictive value (NPV), area under the curve (AUC) and accuracy (AC). The degree of agreement between the ELISA assays using *r*Peroxidoxin, SLbA or the EIE-LVC kit with a parasitological test (biopsy, aspirate or PCR) was determined by the Kappa index (k) values with 95% confidence intervals and interpreted according to the following Fleiss scale: 0.00–0.20, poor; 0.21–0.40, fair; 0.41–0.60, moderate; 0.61–0.80, good; 0.81–0.99, very good; and 1.00, perfect [26]. The one-sample Kolmogorov-Smirnoff test was used to determine whether a variable was normally distributed. For depletion assays, significant differences were detected using unpaired T tests between depleted and undepleted assays. The differences were considered statistically significant at *p*<0.05. All of the statistical analyses were performed using GraphPad Prism (version 5.0) and GraphPad QuickCals (http://www.graphpad.com/quickcalcs/).

## Results

### Prediction of B-cell linear epitopes in peroxidoxin and chemical synthesis of selected peptides

**Figure 1:**
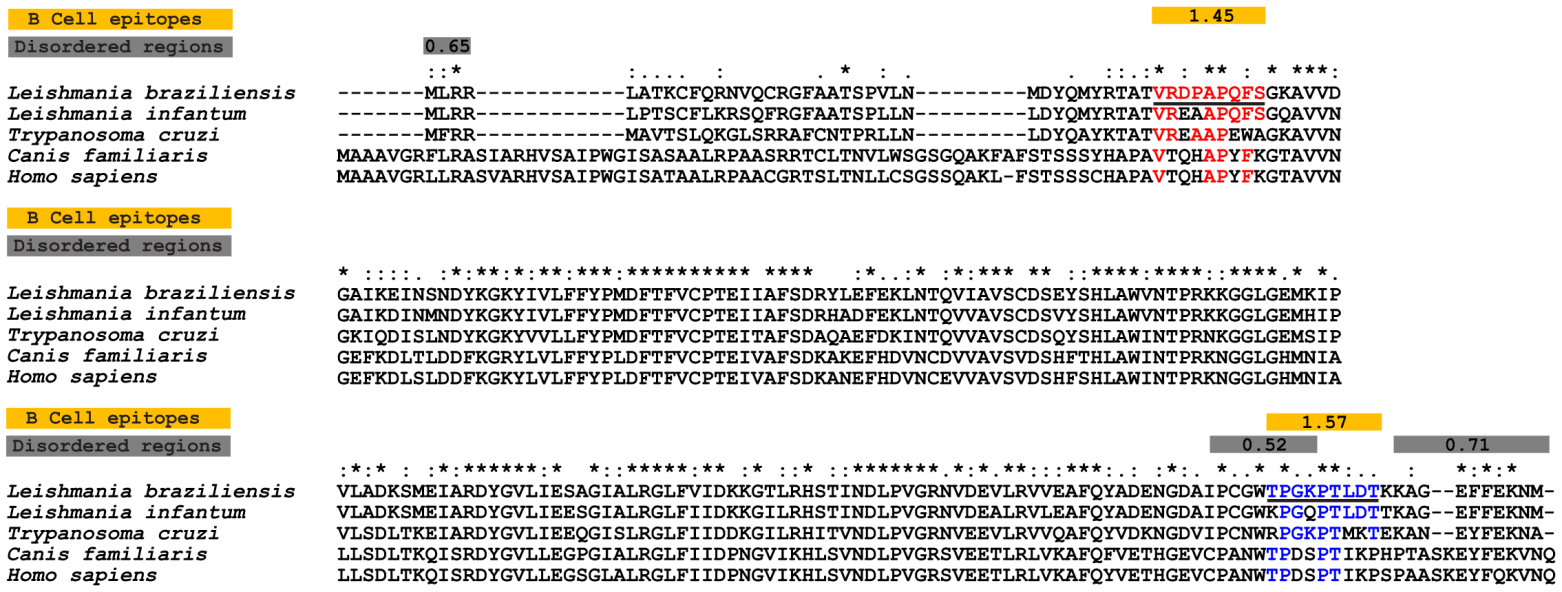
Sequence divergence and prediction of B-cell linear epitopes and intrinsically unstructured/disordered regions in *L. braziliensis* Peroxidoxin and its orthologs. Alignment between *L. braziliensis* peroxidoxin (TritrypDB ID: LbrM.23.0050) and orthologous proteins present in *L. infantum* (TritrypDB ID: Linj.23.0050), *T. cruzi* (TritrypDB ID:TcCLB.509499.14), *H. sapiens* (RefSeq ID: NP_006784.1) and C. *familiaris* (RefSeq ID: NP_001243414.1). The box signifies conserved domains present in the peroxidoxin protein of *L. braziliensis*, as identified by CDD NCBI [48]. The yellow boxes mark predicted B-cell epitopes, and the gray boxes mark predicted disordered regions. The continuous black underlined amino acid sequences represent two potential B-cell epitopes predicted by Bepipred in the LbrM.23.0050 protein, and the colors highlight amino acid conservations in the *T. cruzi*, *C. familiaris* and *H. sapiens* sequences in relation to the *L. braziliensis* sequence.

A gold standard serological diagnostic method is focused on markers that are able to elicit a strong antibody response specific to the pathogen. Although peroxidoxin of *L. braziliensis* has a homologous protein encoded in the genome of *T. cruzi*, *H. sapiens* and *C. familiaris*, polymorphic B-cell epitopes between these organisms may bind to specific antibodies discriminating samples from patients with leishmaniasis. To identify potential epitopes that can increase the specificity of the serological test, we scanned the peroxidoxin sequence of *L. braziliensis* for possible B-cell linear epitopes co-occurring with intrinsically unstructured regions (Figure 1). We observed two predicted epitopes distributed within the protein sequence. The peptide VRDPAPQFS is inside the functional PRX domain, and the other peptide TPGKPTLDT is in the carboxy region. Both peptides were synthesized and used to evaluate their potential for the serodiagnosis of leishmaniasis.

### Expression and purification of the *r*Peroxidoxin protein

**Figure 2:**
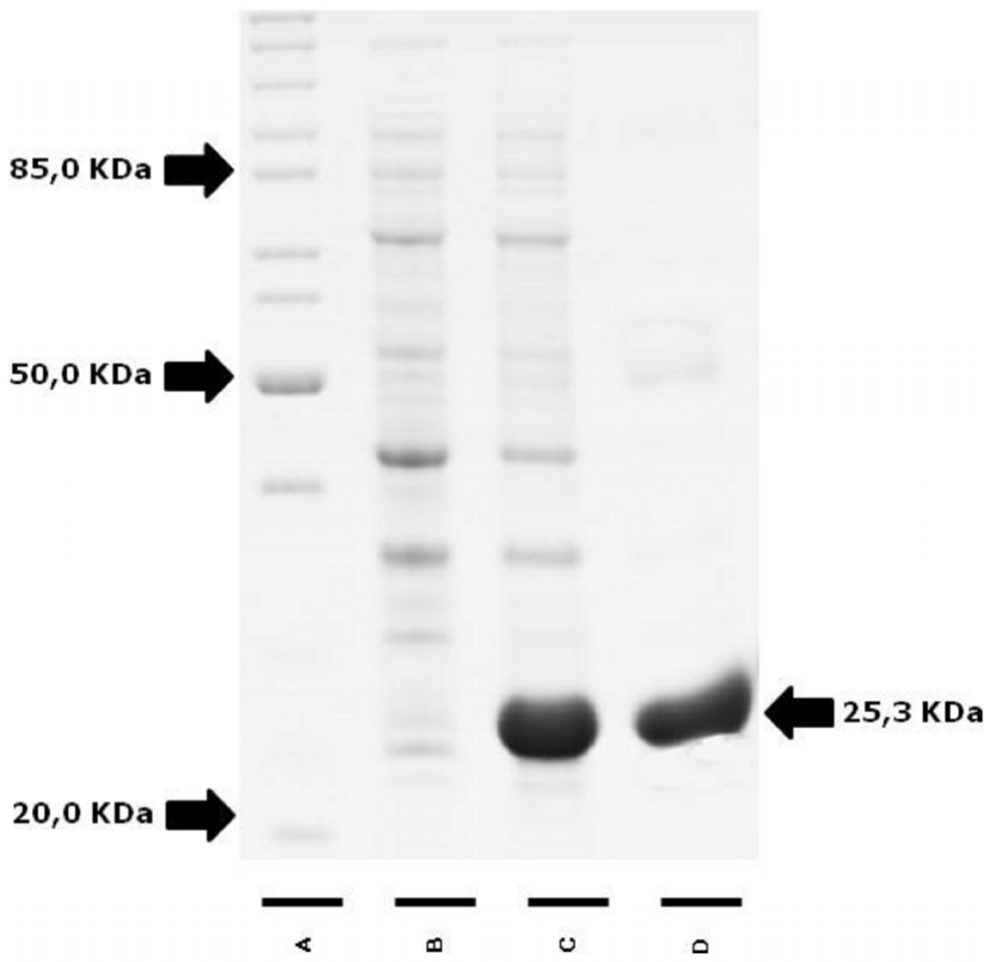
Expression and purification of the recombinant peroxidoxin protein separated by 12.5% SDS-PAGE gel electrophoresis. **(A)** Molecular weight standard, **(B)** lysate of culture before and after **(C)** induction with IPTG and purified protein recombinant peroxidoxin protein (weight: 25.3 KDa) **(D)** by gel filtration.

The full-length coding region of *L. (V.) braziliensis* was amplified by PCR, cloned into the vector *p*GEM and confirmed by sequencing. The gene was then transferred to the expression vector *p*ET28a-TEV, and the *r*Peroxidoxin was overexpressed in *E. coli* BL21 Arctic Express (DE3) in a soluble form. The Ni^2+^-affinity following a gel-filtration column was used to obtain high-purity *r*Peroxidoxin. As shown in Figure 2, the recombinant protein with a predicted molecular weight of 25.3 kDa was successfully expressed and purified.

### Comparison of *r*Peroxidoxin- and SLbA-based ELISAs for TL diagnosis

**Figure 3:**
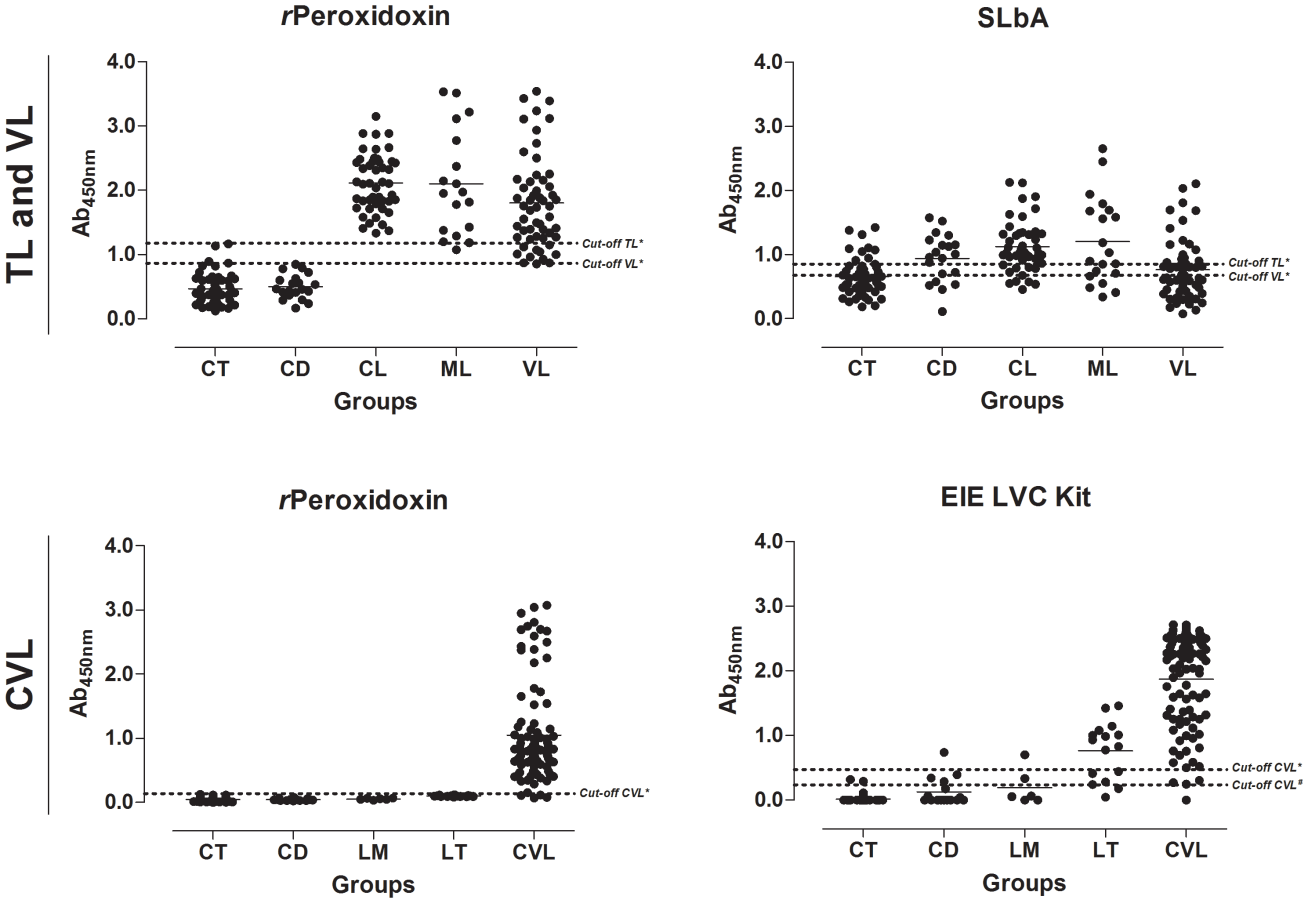
Comparison of reactivity from ELISA against *r*Peroxidoxin, SLbA and EIE-LVC Kit. **TL and VL:** An ELISA was performed in different groups of individuals (CT, control group, n = 50; CD, Chagas diseases, n = 20; CL, cutaneous leishmaniasis, n = 45; ML, mucosal leishmaniasis, n = 20; and VL, visceral leishmaniasis, n = 55). **CVL:** An ELISA was performed in different groups of dogs (CT, control group, n = 51; CD, Chagas diseases, n = 16; LM, Leishmune, n = 6; LT, LeishTec, n = 16; and CVL, canine visceral leishmaniasis, n = 95). ROC curves were used to determine the ELISA cut-off, sensitivity, specificity and AUC.

**Figure 4:**
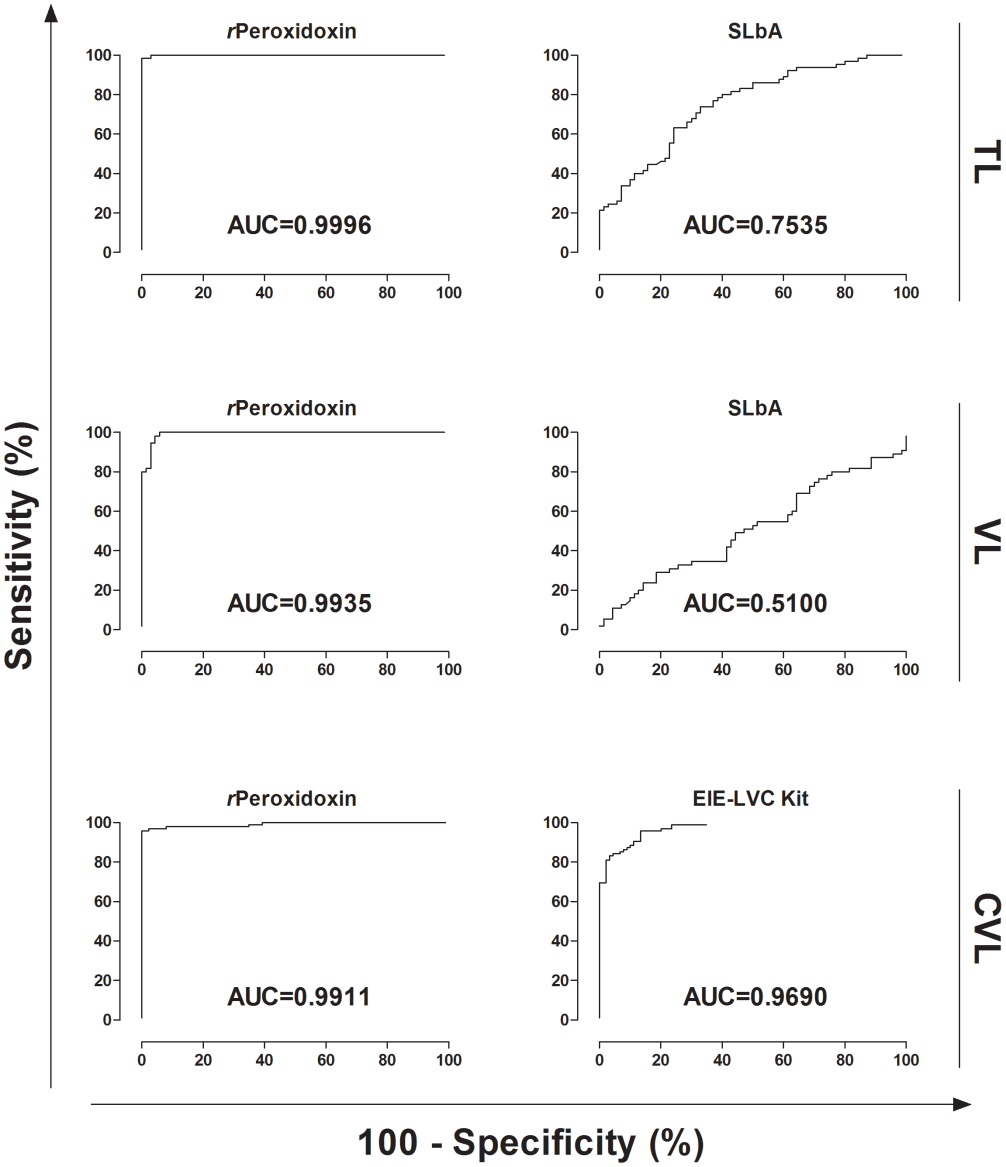
Comparison of ROC curves obtained from *r*Peroxidoxin, SLbA and EIE-LVC Kit. The curves were used to determine the ELISA cut-off, sensitivity, specificity and AUC. ^*^Cut-off obtained by the ROC curve. ^#^Cut-off obtained according to the manufacturer's instructions.

The antigen-specific antibody response against *r*Peroxidoxin in TL was evaluated compared with SLbA ELISA. The data are presented in Figure 3, Figure 4, Table 1 and Table 2. The *r*Peroxidoxin ELISA showed a high sensitivity for detecting the CL (100.00%) and ML (95.00%) clinical forms and presented a total sensitivity (CL+ML) of 98.46% (95% CI: 91.72–99.96%; 1 false negative result in the ML group) for identifying patients infected with *L. braziliensis*. Compared with SLbA, the sensitivity for detecting the CL and ML clinical forms was 84.44% and 70.00%, respectively, showing a total sensitivity of 70.77% (95% CI: 58.17–81.40%) due to 19 false negative results. To evaluate the specificity (Sp), serum samples of chagasic patients and negative control individuals were further tested. *r*Peroxidoxin ELISA demonstrated an excellent specificity value (100.00%; 95% CI: 94.87–100.00%) compared with SLbA (68.57%; 95% CI: 56.37–79.15%), which yielded 22 false positive results. The maximum PPV was achieved by *r*Peroxidoxin (100.00%), followed by SLbA (67.65%), and a high NPV was also observed for *r*Peroxidoxin^#^ (98.59%) compared with SLbA (71.64%). The area under the curve (AUC) and accuracy (AC) were used to compare the efficiency of different diagnostic antigens or tests [25]. *r*Peroxidoxin presented the highest AUC value (0.999; 95% CI: 0.998–1.001), as contrasted with SLb (0.753; 95% CI: 0.673–0.834). The accuracy value for *r*Peroxidoxin was also the highest (AC = 99.26%), as contrasted with SLbA ELISA (AC = 69.63%).

**Table 1:** Measure of diagnostic performance for *r*Peroxidoxin, SLb and EIE-LVC kit.

Test	Disease	Cut-off	Parameters^a^						
			TSe (%)	CI 95%	TSp (%)	CI 95%	PPV (%)	NPV (%)	AC (%)
***r*** **Peroxidoxin** *	TL	1.178	98.46	91.72–99.96	100.00	94.87–100.00	100.00	98.59	99.26
**SLbA** *	TL	0.853	70.77	58.17–81.40	68.57	56.37–79.15	67.65	71.64	69.63
***r*** **Peroxidoxin** *	VL	0.869	98.18	90.28–99.95	95.71	87.98–99.11	94.74	98.53	96.80
**SLbA** *	VL	0.678	52.73	38.80–66.35	50.00	37.80–62.20	45.31	57.38	51.20
***r*** **Peroxidoxin** *	CVL	0.140	95.79	89.57–98.84	100.00	95.94–100.00	100.00	95.70	97.83
**EIE-LVC Kit** *	CVL	0.472	95.79	89.57–98.84	86.52	77.63–92.83	88.35	95.06	91.30
**EIE-LVC Kit** #	CVL	0.237	98.95	94.27–99.97	75.28	65.00–83.81	81.03	98.53	87.50

a Parameters was calculated using all samples presented in this work for TL (CT+CD+CL+ML, n = 135), VL (CT+CD+VL, n = 125) and CVL (CT+CD+LM+LT+CVL, n = 184).

^*^ *Cut-off* obtained by ROC curve.

# *Cut off* obtained according to the manufacturer.

Abbreviations: Tse; total sensitivity; TSp: total specificity; CI: confidence interval; PPV: positive preditive value; NPV: negative preditive value; AC: accuracy.

**Table 2:** Measure of diagnostic performance for *r*Peroxidoxin, SLb and EIE-LVC kit using ROC curves, data validation and agreement using Kappa index.

Test	Disease	AUC	CI 95%	TP	TN	FP	FN	κ^a^	CI 95%	Agreement^b^
***r*** **Peroxidoxin** *	TL	0.999	0.998–1.001	64	70	0	1	0.985	0.956–1.000	Very good
**SLbA** *	TL	0.753	0.673–0.834	48	46	22	19	0.393	0.238–0.548	Fair
***r*** **Peroxidoxin** *	VL	0.993	0.985–1.002	54	67	3	1	0.935	0.873–0.998	Very good
**SLbA** *	VL	0.510	0.405–0.614	29	35	35	26	0.027	−0.147–0.200	Poor
***r*** **Peroxidoxin** *	CVL	0.991	0.979–1.002	91	89	0	4	0.957	0.914–0.999	Very good
**EIE-LVC Kit** *	CVL	0.969	0.947–0.991	91	77	12	4	0.825	0.744–0.907	Very good
**EIE-LVC Kit** #	CVL	NA	NA	94	67	22	1	0.748	0.654–0.842	Good

a Kappa index was calculated using all samples presented in this work for TL (CT+CD+CL+ML, n = 135), VL (CT+CD+VL, n = 125) and CVL (CT+CD+LM+LT+CVL, n = 184).

b Agreement was calculated using parasitological assays as gold standart test.

^*^ *Cut-off* obtained by ROC curve.

# *Cut off* obtained according to the manufacturer.

Abbreviations: AUC: area under curve; CI: confidence interval; TP: true positive; TN: true negative; FP: false positive; FN: false negative; κ: kappa index; NA: not applicable.

### Comparison of *r*Peroxidoxin- and SLbA-based ELISAs for VL diagnosis

The use of *r*Peroxidoxin for VL diagnosis was evaluated and compared with an SLbA ELISA. The data are presented in Figure 3, Figure 4, Table 1 and Table 2. *r*Peroxidoxin showed a high sensitivity for detecting VL and presented a total sensitivity of 98.18% (95% CI: 90.28–99.95%; 1 false negative result) for identifying patients infected with *L. infantum*. Compared with SLbA, the total sensitivity to detect VL was 52.73% (95% CI: 38.80–66.35%) due to 26 false negative results. The *r*Peroxidoxin ELISA demonstrated an excellent specificity value (95.71%; 95% CI: 87.98–99.11%) compared with SLbA (50.00%; 95% CI: 37.80–62.20%), which yielded 3 and 35 false positive results, respectively. The maximum PPV was achieved by *r*Peroxidoxin (94.74%), followed by SLbA (45.31%), and a high NPV was also observed for *r*Peroxidoxin^#^ (98.53%) compared with SLbA (57.38%). The *r*Peroxidoxin presented the highest AUC value (0.993; 95% CI: 0.985–1.002) as contrasted with SLb (0.510; 95% CI: 0.405–0.614). The accuracy value for *r*Peroxidoxin was also the highest (AC = 96.80%), as contrasted with the SLbA ELISA (AC = 51.20%). Similar to that observed for the ELISA assay using SLbA, the test using SLiA (Figure S1) also showed lower performance compared to *r*Peroxidoxin (SLiA: Se = 76.36%; Sp = 75,71; AUC = 0.7761; AC = 76,00%).

### Assessment of the ELISA performance using *r*Peroxidoxin and the EIE-LVC Kit for CVL diagnosis and cross-reactivity in dogs immunized with the Leishmune and LeishTec commercial vaccines

The *r*Peroxidoxin ELISA for CVL diagnosis was evaluated and compared with the EIE-LVC Kit. The data are presented in Figure 3, Figure 4, Table 1 and Table 2. The specificity was also performed in dogs without a history of *Leishmania* infections and that were immunized with the Leishmune and LeishTec commercial vaccines to check for cross-reactivity. In the serological assay, *r*Peroxidoxin showed a higher combined sensitivity and specificity value (Se = 95.79%, 95% CI: 89.57–98.84; Sp = 100.00%, 95% CI: 95.94–100.00) compared with EIE-LVC* (Se = 95.79%, 95% CI: 89.57–98.84; Sp = 86.52%, 95% CI: 77.63–92.83) and EIE-LVC^#^ (Se = 98.95%, 95% CI: 94.27–99.97; Sp = 75.28%, 95% CI: 65.00–83.81). The maximum PPV was achieved by *r*Peroxidoxin (100.00%), followed by EIE-LVC* (88.35%) and EIE-LVC^#^ (81.03%). High NPV values were also observed for *r*Peroxidoxin (95.70%) and EIE-LVC* (95.06%), and the best value was observed for EIE-LVC^#^ (98.53%). In this step, no cross-reactivity was observed for *r*Peroxidoxin (Sp: LM = 100.00% and LT = 100.00%), whereas false positive results were found for the two vaccines through the EIE-LVC* (Sp: LM = 66.67% and LT = 37.50%) and EIE-LVC^#^ (Sp: LM = 66.67% and LT = 12.50%). The *r*Peroxidoxin presented the highest AUC value (0.991), showing a high accuracy (AC = 97.83%), followed by the EIE-LVC* (AUC = 0.969; AC = 91.30%) and EIE-LVC^#^, which, using the cut-off recommended by the manufacturer, yielded a lower accuracy value (87.50%).

### Analysis of the agreement between *r*Peroxidoxin, SLbA or the EIE-LVC Kit with parasitological assays

The agreement (Kappa index) between the *r*Peroxidoxin and SLbA serological tests or the EIE-LVC Kit with parasitological assays is shown in Table 2. Regarding the *r*Peroxidoxin ELISA in the TL diagnosis, very good agreement was observed (0.985; 95% CI: 0.956–1.000) compared with SLbA (fair; 0.393; 95% CI: 0.238–0.548). A very good agreement was also observed in the VL diagnosis for *r*Peroxidoxin (0.935; 95% CI: 0.873–0.998) compared with SLbA (poor; 0.027; 95% CI: −0.147–0.200). When evaluating *r*Peroxidoxin for CVL diagnosis, we observed very good agreement (0.957; 95% CI: 0.914–0.999). For EIE-LVC*, the agreement was lower but still classified as very good (0.825; 95% CI: 0.774–0.907); for EIE-LVC^#^, the agreement was considered good (0.748; 95% CI: 0.654–0.842).

### Synthetic peptides VRDPAPQFS and TPGKPTLDT are recognized by specific antibodies present in the sera of TL, VL and CVL individuals

**Figure 5:**
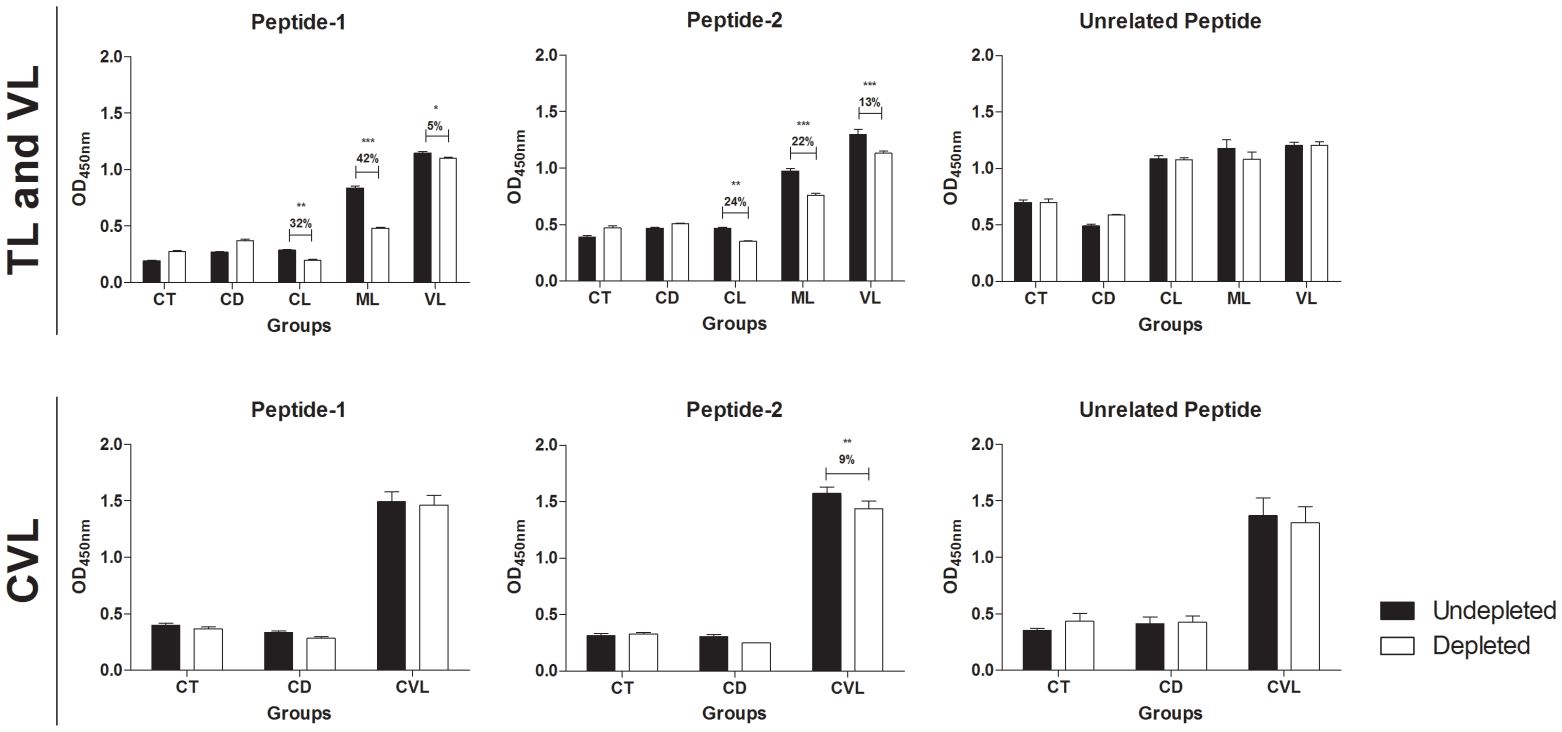
Depletion results showing specific IgG antibody recognition to the synthetic peptides identified in Peroxidoxin. The pool of sera (n = 10) were depleted with peptide-1 (TPGKPTLDT), peptide-2 (VRDPAPQFS) and unrelated peptide (TYHGIPCDSGRNCKKYENF) in different groups (CT, control group; CD, Chagas diseases; CL, cutaneous leishmaniasis; ML, mucosal leishmaniasis; VL, visceral leishmaniasis; and CVL, canine visceral leishmaniasis). The mean antibody OD values are shown on the Y-axis, and the error bars indicate the SD. Significant differences detected using unpaired T tests are indicated on the graphs with significant P values (* *p*<0.05; **p<0.01; ***p<0.001).

To determine the specific recognition of peroxidoxin by TL patients, an ELISA depletion assay was performed using synthetic peptides VRDPAPQFS (peptide-1) and TPGKPTLDT (peptide-2) relative to the predicted linear B-cell epitopes present in this protein (Figure 5). In this assay, the IgG reactivity against *r*Peroxidoxin after the depletion of peptide-1 specific antibodies was reduced by 32% for CL (*p*<0.01), 42% for ML (*p*<0.001) and 5% for VL (*p*<0.05); no reduction was observed in the CVL group. With respect to peptide-2, we observed a reduction of 24% for CL (*p*<0.01), 22% for ML (*p*<0.001), 13% for VL (*p*<0.001) and 9% for CVL (*p*<0.01). Significant differences were not observed in the CD and CT group for both peptides evaluated. No significant differences were observed when unrelated peptide was used in the depletion assay.

## Discussion

The establishment of novel laboratory devices and the validation of alternative methodologies, as well as the discovery of new antigens, still represent a broad investigative field for the accurate differential diagnosis of TL. At present, no gold-standard test for TL exists, and a combination of different diagnostic techniques is often necessary to obtain more precise results [27]. Additionally, the commonly employed parasitological techniques are invasive, time-consuming, and inappropriate for epidemiological surveillance [27].

Serologic tests to diagnose TL may also present limitations such as low sensitivity, specificity, reproducibility, antibody titers and/or the absence of a correlation between the circulating antibody levels with the disease stage. In addition, the tests may be cross-reactive with other species of the Trypanosomatidae family [27]–[29]. In the present work, we present data on the development of an enzyme-linked immunosorbent assay for detecting antibodies against the *r*Peroxidoxin of *L. braziliensis* for potential use in immunodiagnosing TL, VL and CVL compared with soluble *Leishmania* antigen, as well as an agreement analysis with parasitological assays. The goal of this work was to identify antigenic polymorphic linear B-cell epitopes present in this protein that are not recognized by individuals suffering from Chagas disease.

It is well established that proteins stage-specifically expressed in *Leishmania* parasites associated with virulence have a high antigenicity during the active disease phase, which has precipitated the quest to confirm their potential use for diagnosis and as a marker for monitoring the therapeutic response [30]–[32]. Peroxidoxin belongs to a family of proteins called peroxiredoxins (PRXs), which play a vital role in detoxifying reactive oxygen species in the parasites—an activity that is particularly relevant for *Leishmania* [33], [34]. These factors classify PRXs of these organisms as important virulence factors [35], [36], and previous studies have confirmed the antigenicity against these proteins in the sera of human and dogs infected by other *Leishmania* species [30], [37], [38]. In confirmation of these studies, we showed here that TL patients produced high levels of antibody against PRXs during the active cutaneous and mucosal clinical forms of the disease. The high levels of antibodies against peroxidoxin observed in VL and CVL individuals infected by *L. infantum* confirmed the observations in other studies evaluating the antigenicity and immunogenicity against *L. braziliensis* antigens [10], [11], [39]. This effect is due to the high conservation of peroxidoxin among *Leishmania* species, which favors the possibility of cross-reactivity against infection by different *Leishmania* species, thereby broadening the spectrum of diagnosis in dogs present in endemic areas common to other leishmaniases. Similar findings were described by serological assays using orthologous genes of peroxidoxin in *L. infantum*-infected children during acute human VL or in dogs, using immunoproteomic approaches [31], [32].

The antigenicity against peroxidoxin observed in CL and ML groups with mean values of four to five times higher than in the control group, resulted in high values of sensitivity for detecting the disease, including individuals who were MST negative and had a unique presentation and recent cutaneous lesions. In endemic areas, the MST is a decisive method for diagnosing older cutaneous lesions and mucosal lesions, when the number of parasites is low and therefore, difficult to detect [40]. For *L. braziliensis infection*, a low sensitivity value has been previously described by MST when compared with serological methods [41]. The high positive predictive value observed in *r*Peroxidoxin ELISA may represents an alternative complementary assay for TL diagnosis, as well as in the development of a new rapid immunochromatographic test [42]. Accordingly, other studies employing a serological assay in other *Leishmania* species that are part of the *Viannia* complex [43] have demonstrated high positivity in patients with a confirmed diagnosis of cutaneous leishmaniasis with the presence of multiple lesions and in patients with mucosal leishmaniasis when promastigote forms of *L. (V.) panamensis* are used.

Another important aspect of the serologic methods applied to the leishmaniasis diagnosis is the choice of antigen, which still represents a relevant obstacle because it is common to find a large number of false positive reactions observed in individuals infected with other trypanosomatids, mainly when using a crude *Leishmania* antigen due to sharing multiple common epitopes [9], [44]. In our study, when an ELISA was performed using SLbA, high numbers of false positive results appeared in the chagasic patients (65.0%), inclusive and control group (30.0%), which can be explained by the cross-reaction between *Leishmania* and other unrelated diseases caused by common antigenic determinants that hinder a specific TL diagnosis [45], especially in regions where several parasitic diseases are endemic [46]. Previous studies have increased the ELISA specificity in the diagnosis of leishmaniasis through the negative selection of cross-reactive epitopes present in *Leishmania* or *T. cruzi* proteins and synthetic peptides [12], [47]. The large number of false positive results for EIE-LVC might also be related to the antigen used in the assay; the soluble antigen of *Leishmania major*-like presents a greater likelihood of cross reactions with sera from animals infected with other trypanosomatids or protozoa, sharing multiple common epitopes [9], [44].

We found that the peroxidoxin protein presents two polymorphic inmunodominant linear B-cell epitopes, which led us to evaluate the specificity of this protein for diagnosing TL, VL and CVL. Notably, all chagasic and control patients or dogs presented absorbance values below the cut-off established by the ROC curve criterion. The significant IgG depletion observed using synthetic peptides relative to epitopes mapped in TL, VL and CVL confirmed the high specificity of the peroxidoxin ELISA to discriminate between *Leishmania*-infected individuals with CD or CT.

In summary, our data suggest that *r*Peroxidoxin has a promising potential to identify TL, VL and CVL and can be used as an alternative method for confirmation patients with an accuracy above 95%. Additionally, this investigation focused on analyzing the success in employing strategies that previously used bioinformatics approaches to map polymorphic B-cell epitopes present in potential antigens for ELISA to select high antigenic targets and eliminate cross-reactivity with other infections combining the proteomic data of these parasites. Further prospective studies using large cohorts of negative and positive individuals from endemic areas are necessary to better characterize this approach as a possible marker for diagnosing TL, VL and CVL and for monitoring the post-therapeutic cure of TL or VL.

## Supporting Information

Figure S1 (A) Comparison of reactivity from ELISA against SLiA in VL and (B) ROC curve obtained from SLiA: An ELISA was performed in different groups of individuals (CT, control group, n = 50; CD, Chagas diseases and VL, visceral leishmaniasis, n = 55). ROC curves were used to determine the ELISA cut-off, sensitivity, specificity and AUC. ^*^Cut-off obtained by the ROC curve.
(TIF)
