# Supplementary figures and images for: Mapping B-Cell Epitopes for the Peroxidoxin of Leishmania (Viannia) braziliensis and Its Potential for the Clinical Diagnosis of Tegumentary and Visceral Leishmaniasis
Source: PLoS One. 2014 Jun 12;9(6):e99216. doi: 10.1371/journal.pone.0099216 (PMC4055673; doi:10.1371/journal.pone.0099216)

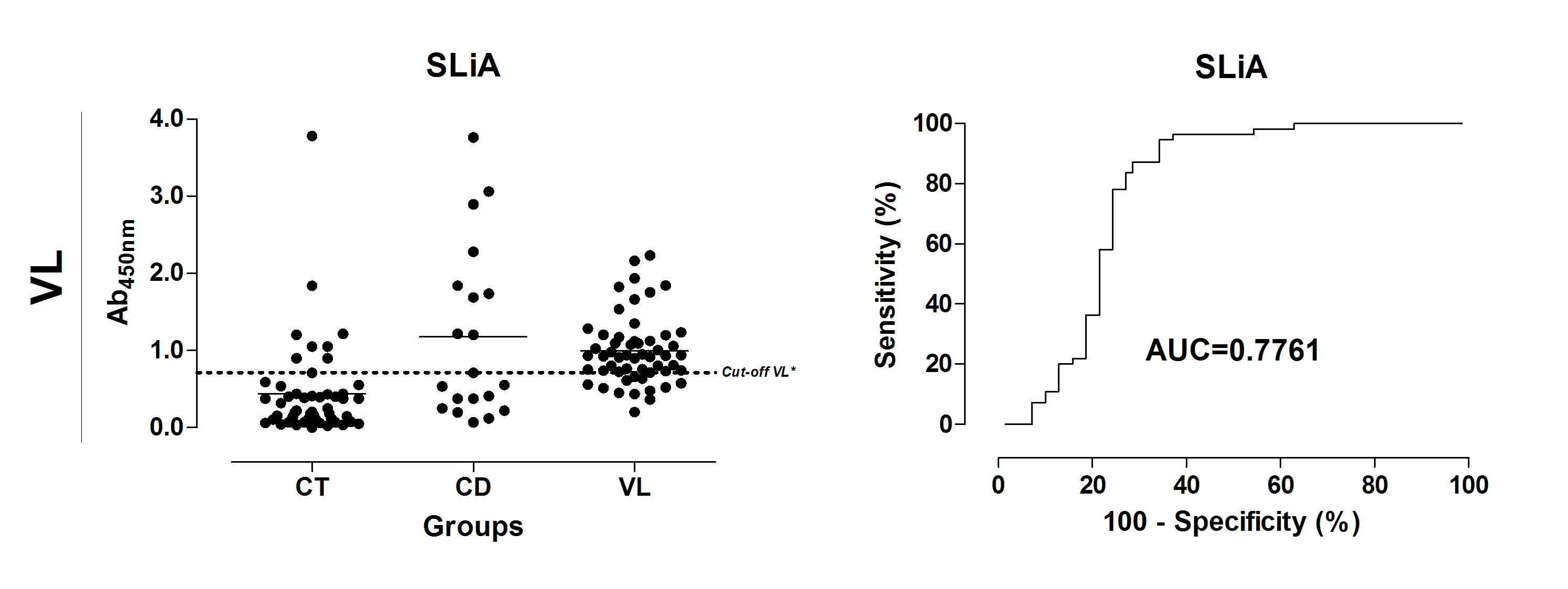

Supplement: Figure S1 — (A) Comparison of reactivity from ELISA against SLiA in VL and (B) ROC curve obtained from SLiA: An ELISA was performed in different groups of individuals (CT, control group, n = 50; CD, Chagas diseases and VL, visceral leishmaniasis, n = 55). ROC curves were used to determine the ELISA cut-off, sensitivity, specificity and AUC. *Cut-off obtained by the ROC curve. (TIF) [file pone.0099216.s001.tif]
